# Pharmacological strategies and suicidal outcomes in borderline personality disorder: observational synthesis

**DOI:** 10.1192/j.eurpsy.2026.11002

**Published:** 2026-07-31

**Authors:** D. Jelaga, M. Belous

**Affiliations:** Department of Mental Health, Medical Psychology and Psychotherapy, Nicolae Testemițanu State University of Medicine and Pharmacy, Chisinau, Moldova, Republic of

## Abstract

**Introduction:**

Borderline personality disorder (BPD) carries high rates of suicide attempts and death by suicide. While psychotherapy is first-line, pharmacotherapy is frequently used for comorbid symptoms; its association with suicidal outcomes remains uncertain and appears class-dependent.

**Objectives:**

(1) Quantify the association between pharmacological classes and suicidal outcomes in BPD; (2) compare effects across classes; (3) outline clinical/policy implications.

**Methods:**

Systematic searches in MEDLINE/PubMed, Embase, PsycINFO and Web of Science. Eligible designs: observational cohorts/longitudinal analyses and systematic/narrative syntheses; randomised trials without suicidal outcomes were excluded. PRISMA: 1,238 records identified; 1,206 after deduplication; 86 full texts assessed; 7 studies included; 79 excluded. Populations: adults with BPD from clinical services and national registries (mean age 28–35 years; 70–85% female). Primary outcome: suicidal behaviour (attempts/death). Secondary proxies: psychiatric rehospitalisation; composite all-cause hospitalisation/death. Adverse events and medication adherence were not consistently reported.

**Results:**

Primary outcome: in a Swedish national BPD cohort (N=22,601; mean follow-up ≈6.9 years), ADHD medication was associated with a 17% lower risk of suicidal behaviour; mood stabilisers showed a non-significant 3% decrease. Antidepressants were associated with a 38% higher risk, antipsychotics with an 18% higher risk, and benzodiazepines with a 61% higher risk of attempts/death; 316 suicides occurred (~1.4%). Secondary proxies: in a second BPD cohort, ADHD medication was associated with 12% lower psychiatric rehospitalisation and 14% lower composite all-cause hospitalisation/death, whereas antidepressants, antipsychotics and benzodiazepines were associated with 17–38% higher risks; mood stabilisers showed no consistent benefit. Confidence intervals for non-ADHD classes were not consistently reported.

**Conclusions:**

Across recent observational evidence, ADHD medication is associated with lower suicidal behaviour (≈−17%) and lower hospital utilisation (≈−12–14%) in BPD, whereas antidepressants and antipsychotics are associated with higher risk (≈+18–38%) and benzodiazepines with larger increases (≈+61%); mood stabilisers show no consistent benefit.

**Disclosure of Interest:**

None Declared

